# Supplementary material for: In-Feed Supplementation of Resin Acid-Enriched Composition Modulates Gut Microbiota, Improves Growth Performance, and Reduces Post-Weaning Diarrhea and Gut Inflammation in Piglets
Source: Animals (Basel). 2021 Aug 26;11(9):2511. doi: 10.3390/ani11092511 (PMC8472311; doi:10.3390/ani11092511)
Supplement: Supplementary file 1 [file animals-11-02511-s001.zip › animals-1336952-supplementary.pdf]

animals

Supplementary Materials

In-Feed Supplementation of Resin Acid-Enriched Composition Modulates Gut Microbiota, Improves Growth Performance, and Reduces Post-Weaning Diarrhea and Gut Inflammation in Piglets

Md Karim Uddin [1,*], Shah Hasan [1,2], Md. Rayhan Mahmud [1], Olli Peltoniemi, [1] and Claudio Oliviero [1]

[1] Department of Production Animal Medicine, Faculty of Veterinary Medicine, University of Helsinki, 04920 Saarentaus, Finland; shah.hasan@helsinki.fi (S.H.); rayhan.mahmud@helsinki.fi (M.R.M.); olli.peltoniemi@helsinki.fi (O.P.); claudio.oliviero@helsinki.fi (C.O.)
[2] Hankkija Oy, Peltokuumolantie 4, 05801 Hyvinkää, Finland
* Correspondence: md.uddin@helsinki.fi

Citation: Uddin, M.K.; Hasan, S.; Mahmud, R.; Peltoniemi, O.; Oliviero, C. In-Feed Supplementation of Resin Acid-Enriched Composition Modulates Gut Microbiota, Improves Growth Performance, and Reduces Post-Weaning Diarrhea and Gut Inflammation in Piglets. *Animals* **2021**, *11*, x. https://doi.org/10.3390/xxxxx

Academic Editor: Paolo Bosi

Received: 27 July 2021
Accepted: 23 August 2021
Published: date

Publisher's Note: MDPI stays neutral with regard to jurisdictional claims in published maps and institutional affiliations.

Copyright: © 2021 by the authors. Submitted for possible open access publication under the terms and conditions of the Creative Commons Attribution (CC BY) license (http://creativecommons.org/licenses/by/4.0/).

Table S1. Ingredient and nutrient composition of sow basal diet and piglet creep feed.

Raw Material (g/kg)	From Weaning to G42	Gestating Multiparous Sows	Transition Period (G110 to L3)	Lactating Multiparous Sows
Dry matter	874	872	874	879
Crude protein	135	115	136	175
Crude fat	30	25	31	42
Crude fiber	46	55	46	37
Ash	56	57	55	63
Calcium	8.2	8.5	8.0	10.2
Phosphorus	5.1	5.1	5.1	5.7
Sodium	2.0	2.0	2.0	2.6

Table S2. Chemical composition of RAC (Progres®).

Fatty Acid Composition %	Weight %
18:2–9,12 Linoleic acid	40.4
18:1–9 Oleic acid	26.9
18:3–5,9,12 Pinolenic acid	6.4
18:2 Conjugated Linoleic acid	6.0
Other fatty acids	9.1
Fatty acids, total	88.8
**Resin acid composition**	**Weight %**
Abietic acid	3.0
Dehydrobietic acid	1.4
Pimaric acid	1.1
Other resin acids	3.7
Resin acids total	9.2

Table S3. Ingredient and nutrient composition of piglet creep feed, post-weaning feed.

Analysis (per kg)	Creep Feed (Control)	Creep Feed (RAC)	Post-weaning Feed (Control)	Post-weaning Feed (RAC)
NE	11.0 MJ	11.0 MJ	9.8 MJ	9.8 MJ
Moisture (%)	12.0	12.0	12.5	12.5
Crude protein (%)	19.0	19.0	16.0	16.0
Crude fat (%)	6.9	6.9	3,8	3,8
Crude fiber (%)	2.9	2.9	4.2	4.2
Ash (%)	5.0	5.0	5.0	5.0
Methionine (g)	5.7	5.7	3.9	3.9
Lysine (g)	15.0	15.0	11.2	11.2
Calcium (g)	7.2	7.2	7.1	7.1
Phosphorus (g)	5.5	5.5	4,8	4,8
Sodium (g)	2.5	2.5	1.7	1.7
RAC (%)		0.1		0.1

NE = net energy: MJ = megajoule. Resin acid-enriched composition (RAC) was a part of the compound feed formulation, added to creep feed, and post-weaning feed by spraying during the feed mixing.

Table S4. Partial Illumina TruSeq adapter sequences added to the 5′ ends.

Forward	Reverse
F_1; CCTACGGGNGGCWGCAG	R_1; GACTACHVGGGTATCTAATCC
F_2; gtCCTACGGGNGGCWGCAG	R_2; aGACTACHVGGGTATCTAATCC
F_3; agagCCTACGGGNGGCWGCAG	R_3; tctGACTACHVGGGTATCTAATCC
F_4; tagtgtCCTACGGGNGGCWGCAG	R_4; ctgagtgGACTACHVGGGTATCTAATCC

Table S5. Abundant taxa in different sows and piglet treatment groups.

Taxa (Phylum)	CCC Mean	RCC Mean	RCR Mean	RRR Mean	*p*-Value
Firmicutes	9.69	9.43	9.52	9.4	0.23
Bacteroidetes	2.07	2.75	2.64	2.87	0.23
Actinobacteria	0.67	0.66	0.65	0.68	0.97
Proteobacteria	0.53	0.98	0.55	0.67	0.0038
Bacteria__unclassified	0.46	0.67	0.48	0.59	0.37
Spirochaetes	0.22	0.61	0.25	0.27	0.0053
Campilobacterota	0.16	0.3	0.18	0.16	0.03
Candidatus__Saccharibacteria	0.14	0.19	0.2	0.16	0.28
Verrucomicrobia	0.085	0.26	0.077	0.11	0.011
Chlamydiae	0.12	0.16	0.15	0.11	0.55
**Taxa (class)**					
Betaproteobacteria	0.075	0.28	0.09	0.099	0.00022
Bacteroidetes__unclassified	0.4	0.95	0.36	0.53	0.0003
Deltaproteobacteria	0.37	0.81	0.44	0.54	0.0015
Spirochaetia	0.22	0.61	0.25	0.27	0.0053
Subdivision5	0.079	0.24	0.066	0.1	0.017
Campylobacteria	0.16	0.3	0.18	0.16	0.03
Erysipelotrichia	1.29	1.23	1.64	1.26	0.084
Bacilli	5.53	5.05	4.48	4.61	0.15
Firmicutes__unclassified	0.54	0.91	0.63	0.71	0.19
Actinobacteria	0.12	0.18	0.013	0.099	0.19
Bacteroidia	2.02	2.56	2.61	2.81	0.26
Candidatus__Saccharibacteria__unclassified	0.14	0.19	0.2	0.16	0.28
Bacteria__unclassified	0.46	0.67	0.48	0.59	0.37
Negativicutes	1.7	1.8	2.14	2.12	0.48
Chlamydiia	0.12	0.16	0.15	0.11	0.55
Clostridia	7.48	7.32	7.76	7.53	0.77
Coriobacteriia	0.65	0.6	0.65	0.66	0.82
Gammaproteobacteria	0.26	0.26	0.23	0.3	0.96
**Taxa (order)**					
Bacteroidetes__unclassified	0.4	0.95	0.36	0.53	0.0005
Desulfovibrionales	0.33	0.78	0.4	0.52	0.0022
Spirochaetales	0.22	0.61	0.25	0.27	0.0053
Campylobacterales	0.16	0.3	0.18	0.16	0.043
Erysipelotrichales	1.29	1.23	1.64	1.26	0.069
Acidaminococcales	0.4	0.43	0.52	0.55	0.091
Selenomonadales	0.61	0.53	0.9	0.78	0.1
Lactobacillales	5.53	5.05	4.48	4.61	0.16
Firmicutes__unclassified	0.54	0.91	0.63	0.71	0.2
Bacteroidales	2.02	2.56	2.61	2.81	0.21
Clostridia__unclassified	0.11	0.16	0.12	0.15	0.23
Coriobacteriales	0.43	0.34	0.45	0.48	0.26
Candidatus__Saccharibacteria__unclassified	0.14	0.19	0.2	0.16	0.31
Bacteria__unclassified	0.46	0.67	0.48	0.59	0.37
Deltaproteobacteria__unclassified	0.13	0.19	0.15	0.14	0.61
Veillonellales	1.44	1.6	1.82	1.84	0.63
Eggerthellales	0.43	0.43	0.4	0.4	0.67
Clostridiales	7.48	7.32	7.76	7.53	0.8
Enterobacterales	0.15	0.16	0.056	0.15	0.87
Coriobacteriia__unclassified	0.19	0.19	0.21	0.19	0.93

**Taxa (family)**					
Streptococcaceae	1.21	0.61	0.21	0.35	0.00015
Bacteroidetes__unclassified	0.4	0.95	0.36	0.53	0.00039
Lachnospiraceae	4.18	3.32	5.23	4.57	0.00043
Desulfovibrionaceae	0.33	0.78	0.4	0.52	0.0022
Bacteroidales__unclassified	0.79	1.4	0.81	0.99	0.0028
Clostridiales__Incertae__Sedis__XIII	0.5	0.73	0.55	0.56	0.019
Ruminococcaceae	3.35	3.65	4.13	4.1	0.021
Erysipelotrichaceae	1.29	1.23	1.64	1.26	0.076
Acidaminococcaceae	0.4	0.43	0.52	0.55	0.12
Clostridiaceae__1	4.25	4.25	3.01	3.32	0.13
Muribaculaceae	0.76	1.03	0.81	0.84	0.13
Selenomonadaceae	0.61	0.53	0.9	0.78	0.14
Firmicutes__unclassified	0.54	0.91	0.63	0.71	0.18
Peptostreptococcaceae	2.08	2	1.69	1.61	0.23
Prevotellaceae	1.65	1.83	2.24	2.36	0.35
Bacteria__unclassified	0.46	0.67	0.48	0.59	0.36
Lactobacillaceae	5.26	5	4.47	4.58	0.52
Veillonellaceae	1.44	1.6	1.82	1.84	0.61
Eggerthellaceae	0.43	0.43	0.4	0.4	0.65
Clostridiales__unclassified	1.27	1.43	1.39	1.41	0.86
**Taxa (*genus*)**					
Bacteroidales__unclassified	0.79	1.4	0.81	0.99	0.0028
Blautia	2.22	1.52	2.7	2.33	0.0018
Butyricicoccus	1.34	1.18	1.75	1.7	0.016
Clostridiales__unclassified	1.27	1.43	1.39	1.41	0.86
Clostridium__sensu__stricto	4.22	4.23	3	3.3	0.14
Dorea	0.86	0.69	0.86	0.8	0.3
Faecalibacterium	1.49	1.18	2.05	1.68	0.054
Firmicutes__unclassified	0.54	0.91	0.63	0.71	0.19
Gemmiger	1.23	0.72	1.42	1.42	0.000067
Holdemanella	0.76	0.43	0.8	0.66	0.0073
Lachnospiraceae__unclassified	2.96	2.37	3.82	3.42	0.0053
Lactobacillus	4.64	4.4	4.1	3.95	0.51
Limosilactobacillus	2.29	2.19	1.76	2.14	0.58
Mediterraneibacter	0.9	0.64	1.18	0.86	0.003
Megasphaera	1.42	1.59	1.75	1.78	0.7
Prevotella	1.51	1.67	2.13	2.25	0.27
Romboutsia	0.72	1.02	0.64	0.57	0.13
Ruminococcaceae__unclassified	1.88	2.64	2.1	2.48	0.053
Ruminococcus	0.84	0.98	1.13	1.03	0.33
Terrisporobacter	1.82	1.58	1.4	1.37	0.2

RRR = sow RAC feeding-piglets RAC creep feeding-piglets post-weaning RAC feeding: RCR = sow RAC feeding-piglets control creep feeding-piglets post-weaning RAC feeding: RCC = sow RAC feeding-piglets control creep feeding-piglets post-weaning control feeding: and CCC = sow control feeding-piglets control creep feeding-piglets post-weaning control feeding.
